# Practical Departments

**Published:** 1906-03-10

**Authors:** 


### PRACTICAL DEPARTMENTS.

### A CHILDREN'S HOSPITAL WITH 200 BEDS.

The Question of Sites.

A correspondent writes: My directors have under consideration a large extension of this hospital, or possibly the complete rebuilding, and with this in view it would be a favour if you would be good enough for their information to refer me to any articles or information in your valuable books on hospitals, dealing with the various points to be kept in view in selecting a site, and erecting a hospital for children, and with the numerous questions regarding construction generally. What extent of ground would be required for a hospital of, say, 200 beds?

. . . It is not easy to fully deal in a short space with these questions, especially as we have no detailed knowledge of your requirements and circumstances. The question of sites is dealt with in Chapter I. of Volume IV. of "Hospitals and Asylums of the World." The article on "The Hospital City," published in The Hospital of December 2, 1905, may also be useful.

If we were charged with the duty of building a modern hospital for town children, to contain 200 beds, we would strongly advise that the hospital should be situated outside the city, and that an ample site should be obtained, to enable the hospital to be built on the one story pavilion plan, with abundance of garden and air space. A well and carefully designed out-patient department, with casualty block containing a few beds, to be erected in some central position within the city, would necessarily constitute an important portion of any such scheme. This receiving house in town would be kept in frequent and rapid communication with the hospital proper outside by a service of motor ambulances of the best design and construction.

Having regard to the most modern developments in treatment, and the results obtained in the treatment of open wounds in cities and outside cities, there can be no doubt, where children are concerned, that every intelligent body of trustees charged with the duty of providing a new hospital for town children must, if they are wise, give their support to proposals modelled on some such scheme as is here briefly indicated.—Ed. The Hospital.

The Charles Urban Biograph at the Alhambra Theatre is exhibiting a series of pictures entitled "What is Whisky," obtained by the courtesy of Messrs. John Dewar and Sons, Limited, and the various processes which the whisky undergoes at the distilleries at Aberfeldy and at the bonded warehouses afterwards.

